# Enhancing measurement of primary health care indicators using an equity lens: An ethnographic study

**DOI:** 10.1186/1475-9276-10-38

**Published:** 2011-09-05

**Authors:** Sabrina T Wong, Annette J Browne, Colleen Varcoe, Josée Lavoie, Victoria Smye, Olive Godwin, Doreen Littlejohn, David Tu

**Affiliations:** 1 University of British Columbia (UBC) School of Nursing, Critical Research in Health and Health care Inequities, 2211 Wesbrook Mall, Vancouver, British Columbia, V6T-2B5, Canada; 2 UBC, Centre for Health Services and Policy Research, 201-2206 East Mall, Vancouver, British Columbia, V6T-1Z3, Canada; 3 University of Northern British Columbia, Department of Community Health Sciences, 3333 University Way, Prince George, British Columbia, V2N-4Z9, Canada; 4 Central Interior Native Health Society, 1110 4th Avenue, Prince George, British Columbia, V2L-3J3, Canada; 5 Vancouver Native Health Society, 449 Hastings Street East, Vancouver, British Columbia, V6A-1P5, Canada; 6 UBC School of Medicine, Department of Family Medicine, 5950 University BoulevardVancouver, British Columbia, V6T-1Z3, Canada

## Abstract

**Introduction:**

One important goal of strengthening and renewal in primary healthcare (PHC) is achieving health equity, particularly for vulnerable populations. There has been a flurry of international activity toward the establishment of indicators relevant to measuring and monitoring PHC. Yet, little attention has been paid to whether current indicators: 1) are sensitive enough to detect inequities in processes or outcomes of care, particularly in relation to the health needs of vulnerable groups or 2) adequately capture the complexity of delivering PHC services across diverse groups. The purpose of this paper is to contribute to the discourse regarding what ought to be considered a PHC indicator and to provide some concrete examples illustrating the need for modification and development of new indicators given the goal of PHC achieving health equity.

**Methods:**

Within the context of a larger study of PHC delivery at two Health Centers serving people facing multiple disadvantages, a mixed methods ethnographic design was used. Three sets of data collected included: (a) participant observation data focused on the processes of PHC delivery, (b) interviews with Health Center staff, and (c) interviews with patients.

**Results:**

Thematic analysis suggests there is a disjuncture between clinical work addressing the complex needs of patients facing multiple vulnerabilities such as extreme levels of poverty, multiple chronic conditions, and lack of housing and extant indicators and how they are measured. Items could better measure and monitor performance at the management level including, *what *is delivered (e.g., focus on social determinants of health) and *how *services are delivered to socially disadvantaged populations (e.g., effective use of space, expectation for all staff to have welcoming and mutually respectful interactions). New indicators must be developed to capture inputs (e.g., stability of funding sources) and outputs (e.g., whole person care) in ways that better align with care provided to marginalized populations.

**Conclusions:**

The current emphasis on achieving greater equity through PHC, the continued calls for the renewal and strengthening of PHC, and the use of monitoring and performance indicators highlight the relevance of ensuring that there are more accurate methods to capture the complex work of PHC organizations.

## Introduction

Over the past decade, decision-makers have made important changes to the organization, financing, and delivery of primary health care (PHC) services, targeting accessibility, continuity, comprehensiveness, and appropriateness of care [1-7]. Interest in renewing PHC is based on solid evidence suggesting that a strong PHC foundation leads to improved population health outcomes, including: reduced risk, duration, and effects of acute and episodic conditions [8-12], as well as reduced risk and effects of continuing health conditions [13-15]. People with access to a regular PHC provider show improved medication adherence [16,17], reduced use of emergency services [18-20], shorter hospital stays [16], and lower overall health care utilization [17].

One important goal of strengthening and renewal in primary healthcare (PHC) is that of achieving health equity [21]. *Equity in health *can be defined as the absence of systematic and potentially remediable differences in one or more characteristics of health across populations or population groups defined socially, economically, demographically, or geographically. *Health inequity *thus refers to differences in health or access to care that can result from structural arrangements that are potentially remedial; in this sense, inequities may be deemed unjust [7]. While these definitions make explicit that health inequities can be measured and tracked over time, debate persists about precisely what should be measured to monitor inequities within the context of PHC service delivery.

There has been a flurry of activity as multiple groups in Canada, the United States of America, and internationally have contributed toward the establishment of indicators relevant to the measuring and monitoring of PHC to account for the impact of investments and to identify areas where service delivery could be improved. Special interest needs to be paid to vulnerable populations [22]. Moreover, more attention needs to be paid to whether current indicators: 1) are sensitive enough to detect inequities in processes or outcomes of care, particularly in relation to the health needs of vulnerable groups or 2) adequately capture the complexity of delivering PHC services across diverse population groups. Current PHC indicators also fall short in terms of capturing the input and outputs that can lead to incremental improvements in health or quality of life that may be possible for people whose health is also affected by systemic and structural inequities. For example, when people live in poverty, lack stable or safe housing, are unable to purchase food on a daily basis, experience the effects of on-going violence (e.g., chronic pain), and/or live with severe mental health and/or substance use issues, current measures may not be immediately relevant or adequate to capture the scope of care required and being provided. Measuring the percentage of women with up-to-date cervical cancer screening as an indicator of preventive cancer care, although important, will overlook the challenges that some groups of women face in accessing services. Challenges include a reluctance to seek preventive care by women with histories of sexual violence and abuse, overcoming barriers related to stigma and discrimination, and a lack of trusting health care relationships required for some women to access even a single visit for health care [23-26]. Alternate or additional measures are therefore needed to capture the complexities and effectiveness of PHC delivery.

**Figure 1:**
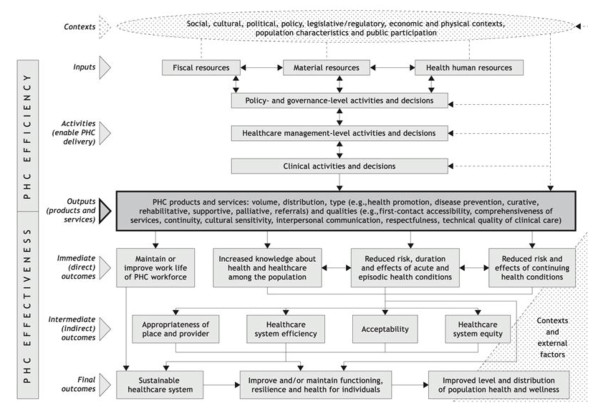
**Results-Based Logic Model for Primary Health Care**. Reprinted with a permission from Longwoods Publishing.

More work is needed to: (i) modify existing indicators relevant to measuring PHC services that are aimed at addressing issues of equity, and (ii) develop new indicators that are sensitive to change, given the complexities inherent in PHC delivery, particularly pertaining to vulnerable populations. The purpose of this paper is to contribute to the discourse regarding what ought to be considered as a PHC indicator and to provide some concrete, practice-based examples that illustrate the need for modification or development of new indicators, given the goal of PHC in achieving health equity. Our intention is that this paper will contribute to the groundwork needed to support such modification and development. The specific aims are to: 1) examine whether elements identified in a commonly used management accountability framework (the PHC Logic Model, Figure 1) is reflected in the care provided in two PHC settings that serve highly marginalized populations, 2) consider whether currently existing PHC indicators reflect the work at non-governmental organizations who deliver PHC to marginalized groups; and 3) provide recommendations relevant in relation to the ongoing work of developing and modifying PHC indicators to better reflect the needs of vulnerable populations.

## Background: Indicators and a Logic Model

In this section, we outline a framework for this work; we describe different types of indicators, and how a management accountability framework, the Primary Health Care Logic Model [27], can be used to identify areas of PHC delivery that could be strengthened. Indicators are standardized measures used to describe population characteristics, community contexts, health status, and health system performance. Indicators, which are identified through some sort of evidence and consensus/consultative process [28,29], can serve different purposes, including system performance and accountability for financial investments. They are designed in part to serve the need of funders to account for the impact of investments in PHC and to also identify areas where PHC service delivery could be improved.

There are three types of indicators: monitoring, performance and developmental, which vary with respect to the kinds of outcomes they can measure. As one moves along the outcome continuum from immediate, to intermediate, to final outcomes, the corresponding degree of attribution from PHC diminishes. In the context of PHC, immediate (or direct) outcomes are those for which this sector is the most (but not solely) responsible and accountable, since these outcomes represent results where PHC has the most control and influence. Even these outcomes, however, can be influenced by external factors and environmental contexts [30].

*Monitoring *indicators are created in areas where different organizational models of practice are 'expected' to have an effect or outcome, but for which attribution is not necessary or possible to demonstrate [30]. Take, for example, a health authority wants to provide better access to mental health services. Indicators are needed to determine where they are succeeding or where they need to improve on. Monitoring indicators are used to recognize that changes in what is being measured may be attributable to a PHC organization's performance and/or other factors. Monitoring indicators can be developed for intermediate or final outcomes. *Performance *indicators are used when it is reasonable to attribute change in what is being measured to an organization's performance. Performance indicators can be used to understand, "How healthy is the health care system?" [31]; these indicators report and account for the organizations' (e.g., solo practice, non-governmental organization, community health centre) inputs, activities, outputs and immediate outcomes [30]. Performance indicators are used to recognize that changes in what is being measured are attributable to the organization's performance more so than any other factor. Not surprisingly, *developmental *indicators are those areas needing development or modification.

Each type of indicator differs in the degree to which performance can be validly and feasibly measured, and in the degree to which results will trigger action. Because performance indicators signal changes attributable to the organization's performance, under an accountability agreement, they can be used to trigger action related to immediate outcomes, but not for intermediate or final outcomes. According to the Auditor General of Canada, accountability is an obligation by government to demonstrate and take responsibility for system performance when measured against targets or goals [32]. Agreements reflect priority setting since they seek to place responsibility on an organization for ensuring that public funding is used and distributed according to the agreed upon purposes. Accountability agreements point to government's desire for increased emphasis on tying an organization's funding to specific deliverables (e.g., performance in quality of care, actual services delivered), while committing the organization to balancing their budget [33].

The PHC Logic Model is one type of management accountability framework which is useful in determining which domains are appropriate for monitoring versus performance indicators. The PHC Logic Model is a heuristic that attempts to visually convey the connection between inputs, activities, outputs, and outcomes [20]. The Logic Model identifies areas for which the PHC sector is directly accountable as well as areas of influence that are external to the PHC sector [30]. The Logic Model suggests that where quality of care (e.g., interpersonal communication) and actual services delivered by PHC organizations and providers has a direct impact (e.g., patient activation), performance can be measured. At this juncture, PHC organizations and providers can be held accountable; therefore, both monitoring and performance indicators can be used. Where PHC organizations have some degree of influence (e.g. health care system efficiency such as avoidable hospitalizations), but where there are also other external factors, only monitoring indicators are appropriate for use.

The PHC Logic Model can assist in identifying areas in which information, evaluation and evidence are needed for policy, administrative and practice communities to plan, monitor, guide and report on PHC renewal [13,27]. This model is currently being used to develop performance indicators that will measure the renewal of PHC in various countries, including Brazil, China, and Canada [27,34].

## Methods

Within the context of a larger ongoing study which examines the delivery of PHC services to vulnerable populations, and in particular, people who are severely impacted by systemic inequities, a mixed methods ethnographic design was used. *Context*. Our research is currently being conducted in partnership with two Urban Aboriginal Health Centers (herein called *Health Centers*), which have been in operation for over 15 years, and which are located in two different inner cities in Canada. Both Health Centers have an explicit mandate to provide health care for Aboriginal people, and to make their services as accessible as possible to both Aboriginal and non-Aboriginal people living with multiple social disadvantages. Many of the patients live on less than $1,000 Canadian dollars (CDN) per month (well below Canada's poverty lines), reside in unstable or unsafe housing, or are homeless. Many of the patients who self-identify as Aboriginal have been affected by the legacy of colonialism (in particular, economic marginalization, discrimination and racism, and intergenerational traumas associated with residential schools and current forms of state child apprehension) [35,36]. The effects of living in poverty intersect with multiple other disadvantages, such as a high proportion of patients experiencing stigma and discrimination related to mental illness, substance use and addictions. Many are affected by violence and have significant chronic pain issues; and increasingly, many people are affected by HIV, AIDS, and related illnesses.

Primary health care services at the two Health Centers are organized around: (a) a primary care medical clinic staffed by physicians, nurses, and nurse practitioners; and (b) outreach and on-site health and social services offered by outreach nurses, addictions counselors, social workers, and social support workers. To varying degrees, indigenous approaches to health and healing [37] underpin the clinics' models of service delivery. We give a brief overview of the larger study that informs the analysis we discuss in this paper, and provide two areas (PHC activities and outputs) as exemplars where indicators could be strengthened.

### Data collection

Three sets of data were collected. At the time of this analysis (2010), these included: (a) participant observation data collected during intensive immersion in the Health Centers (over 850 hours), (b) open-ended, in-depth interviews with Health Center staff (n = 39) who participated in face-to-face individual interviews (n = 29) or a focus group (n = 10), and (c) open-ended, in-depth interviews with patients (n = 68) who similarly participated in face-to-face individual interviews (n = 57) or one of three focus groups (n = 11). Observations focused on the processes of PHC delivery at the Health Centers. The staff interviews included direct care providers (physicians, nurses, social workers, outreach workers and a pharmacist (n = 23) and administrative and support staff (n = 16). Staff interviews focused on the key attributes of service delivery important in the patients' life contexts, how staff members work with patients to facilitate access to health and social services, how continuity of care is established with patients who might otherwise be lost to follow up, and the types of indicators or measures that would be needed to capture the process and impact of providing PHC services. Patient interviews focused on their experiences and reasons for accessing services at the Health Centers and at other health care settings, what was helpful or unhelpful about the services at the Health Centers, and areas for improvement. Staff and patients also provided demographic information. All data were taped, transcribed, and made anonymous. This study was approved by the appropriate ethics institutional review board (University of British Columbia and University of Northern British Columbia) and Memorandums of Understanding were signed between the Health Centers and the research team.

### Analysis

Using the observational data as contextual information, we conducted an interpretive thematic analysis of the interview data using procedures for qualitatively derived data [38-40]. Interview transcripts and observational notes were repeatedly read by the members of the investigative team to identify patterns in the data. NVivo [41], a qualitative computer software package, was used to organize and code the narrative data. As more data were collected and analyzed, coding categories were refined. We then examined the themes expressed in the data in relation to the domains identified in the PHC Logic Model, and in relation to publicly available, existing PHC indicators (e.g. Canadian Institute for Health Information Pan-Canadian PHC Indicators [29]). Because there continues to be data collection as part of the larger study, the analysis discussed in this paper is specifically focused on the provision of a subset of examples pertaining to the domains of PHC that are relevant to consider in relation to indicators in order to answer our specific aims. Credibility of the thematic analysis was continually evaluated with the members of our research team, who included experts in qualitative research, leaders and experts in PHC, and a community advisory committee comprising patient and health care representatives. Triangulation of patient, staff, and observational data also contributed to the rigor and trustworthiness of the analysis [40]. Results are reported for areas where the majority (e.g., 90%-100%) of participants shared the same views.

## Results

Staff had worked in the clinic for an average of four years, and most had college-level or higher preparation (see Table 1). Patients who participated in the study reflected the populations served by the clinics: 50% were women, 75% self-identified as Aboriginal, and 41% had not completed high school. Three-quarters of patients reported they were not currently working. While most patients reported having a place to live (68%), most of these resided in social or low-income housing, with 10% reporting that they lived in a single occupancy hotel room, or at a shelter (10%). Seventy-five percent reported that their lives had been affected by violence (data not shown).

**Table 1:** Participant demographics

Characteristic	Provider (n = 39)	Patient (n = 68)
Clinic site (n)		
1	13	37
2	26	31

Provider position (n)		
Primary care physician	8	-
Primary care nurse	8	
Nurse Practitioner	2	
Pharmacist	1	
Social Worker/PHC coordinator/Case manager	4	
Clinic staff (n)	7	
Medical office assistant/secretary	2	
Alcohol & Drug counselor	1	
Aboriginal support worker	1	
Elder	2	
Office manager	2	
Executive director	1	
Outreach worker		

Age		
Mean (SD)	47.5 (13.0)	46 (8.7)

Gender (%)		
Female	62	50
Male	38	47
Transgender	-	3

Ethnicity (%)		
Caucasian	49	21
Aboriginal	31	75
South Asian	3	-
Asian (e.g. Chinese, Filipino, etc.)	5	-
Other	12	4

Highest Level of Education (%)		
Less than high school	-	41
High School	7	38
College/post-secondary	18	10
Undergraduate	36	3
Graduate studies or more	33	-

Employment Status (n)		
Full-time	12	14
Part-time	17	1
Other	-	2
Not employed	-	51

Number of Years employed at health centre		
Mean (SD)	4.0 (4.0)	-

Our findings suggest that modification of existing monitoring and performance indicators at the PHC activities level is needed if progress toward health equity is to be fostered, measured and achieved. At the Inputs and Outputs level, new indicators need to be developed. PHC activities can be categorized into three types: (a) *policy and governance-level *activities and decisions (e.g., financing and regulation), (b) *management-level *activities and decisions (e.g., hours of operation, use of teams) and (c) *clinical-level *activities and decisions that support outputs (e.g., the degree to which clinicians elect to specialize in specific types of clientele such as marginalized groups) [42]. Our analysis focuses on examples of management and clinic level activities because the data are mostly of these kinds.

### Suggested Modification of Existing Monitoring and Performance Indicators

#### PHC Activities: management level

As shown in Table 2, existing objectives and indicators for equity-oriented performance measurement are broad in relation to management level activities, including items such as: "specialized programs for PHC vulnerable/special needs populations," "support for PHC vulnerable/special needs populations" and "PHC family physicians/general practice physicians/nurse practitioners working in interdisciplinary teams" [29]. Our analysis suggests that it is not merely the presence or absence of such programs and approaches that matters; rather, measuring *what *is delivered and *how *it is delivered can contribute to increasing the effectiveness of PHC for marginalized groups. We observed and heard from the majority of providers about how the team approach is enacted to provide a wide range of services. For example, at each site, weekly meetings including the full inter-professional team (providers and support staff) are held to discuss the complexity of care needed by clients. There is an intentional flattening of hierarchical relationships that can arise between professional groups that are typically imbued with different levels of power. Effort is intentionally invested in creating respectful interactions among staff, and all information is valued, regardless of professional hierarchies. One office staff respondent explains why s/he finds it professionally rewarding to work at the Health Center:

**Table 2:** Examples of the need to modify or develop PHC indicators: Inputs, Activities, Outputs

PHC Logic Model	Examples from Pan-Canadian PHC Indicators (CIHI)	Study recommendations
Input-Fiscal Resources	Objective: Provider payment methods that align with primary health care goals -PHC provider remuneration method -Average PHC provider income by funding model	**Recommended Areas for Development of New Indicators** -source(s) of funding -stability of funding
Activity-Management level	Objective: To increase the number of PHC organizations who are responsible for providing planned services to a defined population: - PHC outreach services for vulnerable/special needs populations - Specialized programs for PHC vulnerable/special needs populations - Support for PHC vulnerable/special needs populations	**Suggested Modification of Monitoring and Performance Indicators** -Increase operability of currently available indicators to elucidate *how *PHC organizations can successfully deliver PHC services to vulnerable/special needs populations: -weekly team meetings of all clinic staff -collaboration and input from all clinic staff on care plan and management -number and type of places where care is delivered (e.g., clinic, home, street) -supportive environment where management rewards respectful interactions between all staff -supportive environment where patients feel comfortable
Activity-Clinic level	Objective: To facilitate integration and coordination between health care institutions and health care providers to achieve informational and management continuity of patient care -Use of standardized tools for coordinating PHC -Collaborative care with other health care organizations -intersectoral collaboration -PHC team effectiveness	-number of patients receiving assistance for housing, food stamps, obtaining welfare -number of patients who have charts with trauma history recorded -Use of appropriate skill mix (e.g., physician, nurse, social worker, drug and alcohol counselor, elder) to provide complex PHC -Support for individual staff to develop and enhance respectful communication amongst staff and patients (e.g. time for critical self-reflection, opportunities for providing/receiving support feedback)
Output-quality: Whole Person Care	Objective: To enhance the provision of whole-person comprehensive PHC services, including episodic and ongoing care with increased emphasis on health promotion, disease and injury prevention and management of common mental health conditions and chronic diseases: - Scope of PHC services - Health risk screening - Smoking cessation advice in PHC - Alcohol consumption advice in PHC - PHC initiatives for reducing health risks - Smoking rate - Fruit and vegetable consumption rate - Overweight rate - Heavy drinking rate - PHC resources for self-management of chronic conditions - Time with PHC provider - Client/patient participation in PHC treatment planning	**Recommended Areas for Development of New Indicators** **-**Assessment of individual's social environment -Assessment of individual's emotional health -Treating individual as a person (not a case or a disease)

"I also think that the fact that it's a level playing field for everybody that works here makes such a difference. It's just everybody is considered on the same level, doctors, executive directors like the bosses are, we know they're the bosses but they're also somebody that can sit beside you and do the same job. You know, it's a level playing field...It's hard to describe, you've been in our meetings, have you ever been to one of our meetings in the mornings? That is to me what keeps the life, the heartbeat going in this place. Because we all have the same goal, you know, client care, and it shows in everyone and we all have input which is awesome." [HC #16]

Staff members are expected to actively contribute their input into how best to provide care. For example, medical office assistants (staff who act as receptionists, do scheduling with patients, maintain health records, etc.) routinely have important insights to contribute regarding both particular patients, and the processes of care. There is an underlying expectation that the responsibility of care lies with the team (rather than with the patient or with a single provider), that each staff member has a role, and that, although patients may have strong long term relationships with particular providers, more than one health care provider works with the client to manage his or her care.

Management level decisions intentionally create an environment that fosters and nurtures respect for patients as a strategy to increase accessibility and to optimize responsiveness to a range of intersecting health issues; this stands in sharp contrast to other types of organizations offering primary care services in the same geographic region (e.g., walk-in clinics, solo practice offices) that often specify that only one concern can be addressed at each consultation. We observed the Health Centers' purposeful and locally relevant creation of welcoming atmospheres through the effective use of space, and expectations that most staff have welcoming and mutually respectful health care interactions with all patients, even when some patients' behaviors may be viewed as challenging (e.g. under the influence of alcohol or drugs). The physical spaces are tailored to target the populations served. For example, because the centers serve many people who lack stable, safe housing and who are living in inadequate spaces and places, the entrances (layout, appearance, situating of staff) are designed to encourage people to escape from the cold and to socialize with other patients over a cup of coffee and/or use the phone and/or computer, regardless of whether they have a health care appointment or not.

The importance of enacting welcoming strategies for people experiencing continuous social stigma and discrimination, including racism in health care, cannot be underestimated [43]. The majority of patients repeatedly described how these efforts resulted in a level of comfort and increased willingness to access care. The following quote from a patient illustrates how the environments of the Centers serve to increase access to care:

"You just come in and you feel like right at home, like you can just, like you know everybody and everybody knows you. You don't have to sit in a room like in a doctor's office and be real square, really uptight. Here, it's like you see people walking back and forth, conversations happening all the time. It's like you're a piece of this place, you're not just a number. It's like a home." [PT #13]

#### PHC Activities: clinic level

Findings from our study show that clinic level decisions by staff take into account broader social determinants of health, rather than primarily focusing on medical health service delivery as is typical for the majority of primary care practices. Given the complex health and social issues for these patients (e.g., lack of safe or stable housing, histories of trauma, interpersonal violence, mental health issues, substance use and addictions, HIV and other serious chronic illnesses, poverty, inability to afford adequate food, etc.), a purely biomedical approach to "treating" present medical issues in isolation of social influences is insufficient. Rather than organizing to provide a particular service (e.g., an immunization program, an anger management program), clinic level decisions are oriented to providing the appropriate mix of skill and expertise to meet the intersecting needs of those served. Staffing decisions at the Health Centers are designed to involve a team of experts, including: social workers, drug and alcohol counselors, and outreach workers, to tailor PHC services in ways that address the complex social determinants of health and the consequences of those influences (malnutrition, homelessness, mental health issues, substance use and addiction). Multidisciplinary teams, for example, are mobilized to enact action plans to support pregnant women who are at risk of having their children apprehended by the state. The staff worked to help these women find safety away from domestic violence, obtain safe, stable housing, possess adequate amounts of nutritious food, and provide access to prenatal care, counseling, and parenting support. They also coordinate with child protection services to create effective working relationships and optimize parental contact and involvement. At both Health Centers, women who have known histories of substance use are particularly supported.

Consistent with both Health Centers' mandates to make services as accessible as possible, staff members are expected to actively convey an accepting attitude toward patients in a manner that conveys respect both through their spoken words and non-verbal communication. Given the high proportion of Aboriginal people in the local areas and the aims of the Centers, Aboriginal art and welcoming signs (in one site in a local indigenous dialect) convey respect for the cultural heritage of Aboriginal people and their communities. As one provider points out:

"I think it's a balancing act between appearing professional and knowledgeable and capable...and addressing people in a friendly manner and very often using first names...I think one of the important things when you are consulting with a patient is small talk...we'll talk about experiences in a patient's life that I know about...showing interest in a patient as a whole person as opposed to a list of diseases." [HCP #8]

Our study showed that engagement in what may seem like "small talk" held particular significance for most patients, all of whom are often dismissed or treated in an abrupt manner in everyday social interactions. These social processes and ways of communicating have an important impact on healthcare access. For example, staff reported that many patients attend the Health Centers' outreach or drop-in activities (e.g., women's wellness program or drop-in lunches) for months before accessing primary care providers. Staff also worked to create this same environment outside the Health Center walls. Center physicians and outreach workers from various disciplines routinely see their patients when they are hospitalized, thus modeling respectful, safe interactions in an institutional setting. Staff also worked to enhance positive attitudes, understanding of, and action on the marginalizing conditions patients face in the wider health and social service sectors and the general public. For example, staff in both settings work on community housing initiatives. Finally, providers are afforded the flexibility of delivering PHC services on a drop-in basis, even outside of the clinic schedule, if needed. One provider commented, "...you know it's got to be flexible and you've got to be able to live with chaos..." [HCP #4]. These management decisions and resulting work processes have had a positive impact on the subsequent outputs and outcomes. The services, and the way in which these services are provided, produce outputs such as continuity of care and comprehensiveness of services, as this health care provider describes:

"...the amount of interactions that our patients have with health care professionals has greatly increased since we have been here...maybe they spent long hours in the emergency department prior to us being here...if patients feel very comfortable coming here and just saying, "can I talk?"...their connection with health care professionals is much stronger now...if we weren't here they wouldn't have access to these services or they wouldn't know where to start." [HCP #12]

To summarize, some publicly available indicators likely require greater attention to the measurement of what is being delivered and how it is being delivered. Our results suggest areas (Table 2) where gaps currently exist in operationalizing monitoring and performance indicators within the area of PHC activities at the management and clinic level. Examples of monitoring indicators at the management and clinic level that would be important for increasing health equity include: weekly team meetings including all clinic staff, number and type of places where care is delivered (e.g., clinic, home, street), and number of patients who have charts with trauma history recorded. Importantly, more work is needed in relation to what should be measured as monitoring and performance indicators.

### Recommended Areas for Development of New Indicators

#### Inputs: fiscal resources

An exemplar of where new indicators are needed is in the area of inputs, or Fiscal Resources. In the PHC Logic Model, this domain refers to funding of the organization. What is unique about the Health Centers is that they are both not-for-profit non-governmental organizations with mandates to deliver PHC. They receive and obtain funding from various sources. Our observations of the Health Centers' sources of funding suggest that the amount of funding, the source of funding, and the stability of these funds over the long term has implications for the organizations' ability to hire appropriate staff and for the health services offered. Currently there are no indicators for measuring fiscal resources in the PHC sector, aside from absolute counts of dollars received by PHC organizations. Indicators are also needed to measure the sources of funding, the focus of funding sources (appropriateness) and the stability of these funds [44].

#### Outputs: quality, whole person care

Another exemplar of where new indicators need to be developed is in the area of outputs, or whole person care. Upon closer examination of how it is currently measured (see Table 2), whole person care is equated with the provision of a comprehensive range of PHC services [29]. Current indicators for measuring whole person care focus on whether or not different services are provided by PHC organizations, such as "health risk screening" and "smoking cessation advice in PHC;" however, our analysis illustrates that such screening of advice is likely to be ineffectual unless broader contexts are taken into account. This suggests that whole person care should be defined more broadly, aligning more with Haggerty, et al.'s definition, "The extent to which a clinician elicits and considers the physical, emotional, and social aspects of a patient's health and considers the community context in their care" (p. 340) [45]. Indicators are needed to measure the physical, emotional, and social aspects of a person within the context of his/her community. In relation to equity oriented PHC, care processes need to take into account factors such as: the root causes of chronic conditions, such as chronic pain; challenges associated with meeting basic needs for shelter, food, and a safe living environment; and experiences of everyday discrimination and stigma, and the impact on health and access to health care.

Findings indicate that whole person care is illustrated when the provider not only takes into account the necessary medical tests, procedures, and treatments, but also the person's emotional and social aspects. As two patients describe:

"It's like I'm not better than them, they're [health care providers at the clinic] not better than me and it's okay to talk about it." [PT #01]

"That's one of the reasons why I come here is I just feel comfortable. I come here and do my blood work because I was a [drug] user...it's a trigger for me coming just to get my blood work...people here know how to deal with the veins, they know how to deal with all the scars and all that crap...that's why I come here. I feel comfortable and they offer so much, I've got my HIV services right here...I have everything there, I have counselors...I usually see the doctor, and they just offer everything." [PT #05]

We repeatedly heard from the majority of patients that the approach had profound effects, as this patient describes,

"That's how they support me, keep me on my feet and keep me positive thinking...I feel very safe with them [Health Center Staff]. If they were not here, I think I would be right on the junk [drugs]... I feel so comfortable [here]..." [PT #09]

The majority of providers noted that when the general milieu and non-verbal communication were dismissive, care likely would be discontinuous, even when medical services were provided, because patients would not return for needed health services. Patients and providers discussed that addressing patients as people by "checking in" and saying, "it's good to see you," "thank you for coming in," or "I'm glad you've come back again" [HCP #10], and letting patients direct their care is integral to achieving better care outcomes:

"Sometime, you know, people aren't here to talk about their smoking.....or lots of times it might say "pap test" on my daybook, but the person gets in here and they've just had a fight with their boyfriend and they've been kicked out of their house...had a bunch of triggers to go and use [illicit drugs] and they've tried not to and they are involved with the ministry [because their children are either wards of the province or he/she is being monitored for their parenting abilities]... and sometimes [a visit] can go down a completely different road...They [patients] do need their usual primary care indicators done right...you know, their lipids, A1C, pap, or whatever...you know it's always this balance of your agenda and the patient's agenda and how do you mix those things together. I think that is how we would achieve better care, being able to balance the provider agenda with the patient agenda." [HCP #23]

Our analysis suggests that new and expanded items are needed to more fully measure whole person care. Examples of possible items include: taking into account a person's social environment (e.g., lack of income for maintaining adequate food, or unsafe housing) and recognition of the patient as a person. Such items would reflect a broader definition that recognizes a person's physical, emotional, and social aspects of her/his life and the environment in which s/he lives in. A provider's ability to form a therapeutic relationship with patients and subsequently influence health outcomes could be strengthened through improving the provision of whole person care.

## Discussion

Primary health care system effectiveness in delivering services and its role in helping the health system achieve the goal of equity continues to evolve. While the PHC Logic Model framework is reflected in the care provided by these two Health Centers serving highly vulnerable groups, continued work on measurement for the purposes of monitoring and performance is needed. Similar to findings at Canada's most recent Health Indicators Consensus conference [46], our results suggest that currently available PHC indicators continue to fall short of alignment with the goal of equity, and that more work is needed. Our results suggest that currently existing PHC indictors do not reflect important work that organizations serving vulnerable groups are carrying out and that new knowledge from a variety of sources (e.g., clinicians, patients, decision-makers) and models of PHC delivery (e.g., non-governmental organizations, community health centers, group practices, solo practices) are needed. In some areas, such as activities at the management level, better operationalization of indicators is needed. In other areas, new indicators ought to be introduced based on a broader and agreed upon conceptualization of the construct. Other ongoing research similarly suggests there is a particular lack of fit between some of the existing PHC indicators and relevant key attributes of service delivery in the context of socially disadvantaged people's lives [45].

Examination of current PHC indicators suggests that the notion of "vulnerable populations" has been constructed as a somewhat static concept [or label] applied to particular groups of people. However, research continues to show that the conditions that lead to vulnerability are dynamic and shifting, and that vulnerability along a number of dimensions can be experienced by anyone, depending on their circumstances, history, and life opportunities [47,48]. Measuring performance should not simply be whether or not programs are offered to vulnerable groups, but rather, *what *is offered in terms of PHC activities and outputs. Structural conditions, such as lack of social housing, a minimum wage that lags far behind the cost of living, increasing restrictions on eligibility for social welfare, and social welfare payments that fall well below the poverty line profoundly influence health and access to health care. As Starfield suggests, overall improvements to equity in health will likely require generic interventions aimed at the person [and populations] rather than ones aimed at specific manifestations of illness (such as substance use, anger management, disease) [7].

A core set of PHC indicators could be developed, for both monitoring and performance measurement purposes, as defined by provincial and/or federal jurisdictional mandates. For example, funders focused on increasing health system equity may require a specific set of core indicators that requires particular attention be paid to vulnerable populations or to how the social determinants of health can be addressed in the process of delivery PHC services. It is also likely that at the organizational, or practice level, some PHC indicators may be considered monitoring and performance indicators, whereas for other organizations, these same indicators may be considered only monitoring indicators. What is considered monitoring versus performance indicators will depend on the accountability agreement between the funder(s) and the respective PHC organization. For example, some organizations clearly have a mandate for delivery of PHC services for populations made vulnerable due to intersecting determinants of health such as poverty, lack of housing and no social support. For these types of organizations, performance indicators may focus on *how *the organization delivers PHC services, including an intentionally flat management hierarchy and weekly team meetings. That is, indicators of performance for these types of organizations may have less focus on preventive indicators such as "smoking cessation advice" or "fruit and vegetable consumption rate," and more focus on whether life conditions conducive to smoking cessation can be fostered, whether food can be purchased, and whether the person has a place to prepare food.

Using both the PHC Logic Model and the results from our data underscore the complexity of delivering PHC generally, and more specifically to populations who are most affected by systemic and structural inequities. PHC organizations and providers need to deliver high quality technical and interpersonal care as well as share a philosophical commitment to social justice and fostering equity in the everyday provision of PHC, including approaches that take into account the social determinants of health. Moreover, mobilization, at the management level, of additional resources is needed to move towards more equitable PHC service delivery (e.g. use of a PHC team that includes social worker, mental health counselor, not just clinicians). Our work suggests that tailoring of PHC programs, support services, and outreach is important to the delivering of services that address the multiple complexities of PHC among various population groups.

This work is limited in that the data were collected in two urban Health Centers in Western Canada that serve populations who are severely impacted by systemic inequities, and who face multiple complexities in their everyday lives. We focused solely on management and clinic level indicators, thus, further work is required at other levels. Future work in refinement of PHC indicators needs to be informed by both population health and health services research frameworks. Population health frameworks focus on ecologic or multilevel determinants such as cultural, community, social, environmental, and other contextual factors [49]. Health services research frameworks can guide the development of processes of care that can influence quality, including both technical and interpersonal processes of care, such as whole-person care. Indicators need to be developed that take into account organizational, administrative, and clinical determinants of care.

## Conclusions

Despite these limitations, our study clearly illustrates that more work is required to develop indicators that adequately capture the complex work of PHC. Ongoing dialogue and work is needed by clinicians, PHC organizations, and decision-makers regarding what can be used to capture the work of PHC. Moreover, simply measuring PHC organizations on reaching specific clinical target rates does not take into consideration the complexity of peoples' lives, or the interdisciplinary and complex nature of care that is mobilized to respond to peoples' diverse needs. Sophisticated analytic techniques and the incorporation of newer measures (e.g., Canadian Index of Wellbeing [50] and Indicators of Health Inequalities [51]) are also needed. Given that performance is mainly based on what is measured, and that improvement in performance will be driven by what is currently measured, more work is needed to fully develop organizational and interpersonal indicators to capture work at this level in order to ensure adequate funding, involve a proper mix of provider expertise, and address the social determinants of health. The current emphasis on achieving greater equity through PHC, the continued calls for renewal and strengthening of PHC, and the use of monitoring and performance indicators highlight the relevance of ensuring that there are more accurate methods to capture the complex work of PHC organizations.

## Competing interests

The authors declare that they have no competing interests.

## Authors' contributions

All authors conceived of the study, participated in its design, and developed the initial draft of this manuscript. SW, AB, CV, JL, & VS led all aspects of data collection, literature reviews, and collaboration with our clinical partners. OG, DL, & DT participated fully in the analysis and interpretation of the results. All authors were involved in analysis of data. All authors read and approved the final manuscript.
